# Potential Energy Curves of Core-Excited States of the U M_5_ Absorption Edge Manifold of UO_2_^2+^

**DOI:** 10.1021/acs.inorgchem.5c04776

**Published:** 2026-02-19

**Authors:** Robert Polly, Paul Bagus

**Affiliations:** † Karlsruher Institut für Technologie (KIT), Campus Nord, Institut für Nukleare Entsorgung (INE), Hermann von Helmholtzplatz 1, 76344 Eggenstein-Leopoldshafen, Germany; ‡ Department of Chemistry, 3404 University of North Texas, Denton, Texas 76203-5017, United States

## Abstract

The X-ray Absorption Near Edge Structure of the U M_5_ X-ray absorption edge of UO_2_^2+^ is analyzed using the potential energy curves of an isolated UO_2_^2+^ obtained from rigorous, multiconfigurational all-electron *ab initio* wave functions for the ground and core-excited configurations. The spectroscopic parameters for the potential energy curves are reported. Two novel theoretical methods have been used as measures of the covalent character of the U–O bond: (1) The projection of the U­(5f) and U­(6d) orbitals of the isolated U^6+^ cation on the orbitals of the ground and the core-excited states of UO_2_^2+^ and (2) the size of the orbital charge distributions given by the ⟨*z* ^2^⟩ expectation values. This gives direct insight into the variation of the electronic structure of the bond as the U–O bond length is changed. The excellent agreement of the simulated spectrum with the experimental U M_5_ XANES spectrum proves the validity of our theoretical model. The potential energy curves and the simulated XANES spectra for varying bond lengths together with the information from the two measures about the covalent character allow us to establish a link between X-ray spectroscopy and chemical bonding.

## Graphical abstract

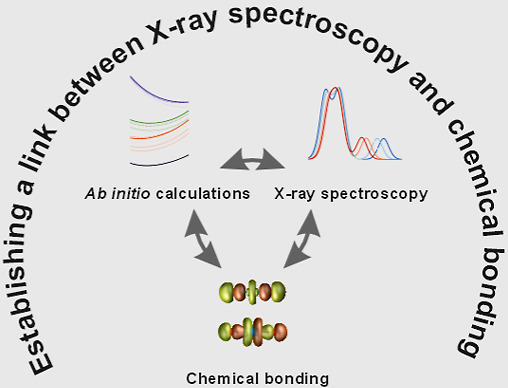

## 1 Introduction

Actinides are important, especially because of applications for nuclear energy production and for nuclear waste disposal.^1–3^ In particular the hexavalent linear actinyls, AnO_2_^2+^ are interesting because these actinyls are found in many different ligand environments.^4–6^ In the present paper, the X-ray Absorption spectra, XAS, is investigated for one of these actinyls, uranyl, UO_2_^2+^ based on theoretical studies of the electronic structure as a means of obtaining a rigorous characterization of the chemical bonding in this compound. For an overview about the current development in theoretical chemistry concerning the calculation of X-ray spectra of actinide compounds. we refer the reader to the representative references in.^7–13^

The uranyl UO_2_^2+^ U M_5_ absorption edge, 3d_5/2_ → 5f_5/2_ and 3d_5/2_ → 5f_7/2_, X-ray absorption near edge structure (XANES)^14^ spectra, denoted as *M*_5_ → 5f, show distinct sets of features which usually contain unresolved excitations to excited states that have nearly the same energy.^15,16^ Different excitation energies arise from the splitting of the seven U­(5f) open-shell orbitals due to the spin–orbit (SO) and ligand field (LF) splittings in uranyl (see Figure 1). The energetic splitting of orbital energies due to SO coupling is ≈1 eV, and due to the LF splitting it is ≈6 eV. Since the 5f valence orbitals form covalent bonds with the oxygen ions, they contain important information which can be probed with X-ray excitations, as, for example, with U M_5_ edge XANES. The LF splitting of the 3d orbital energies is very small;^10^ negligible for the assignment of the features in the *M*_5_ → 5f XANES.

**Figure 1:**
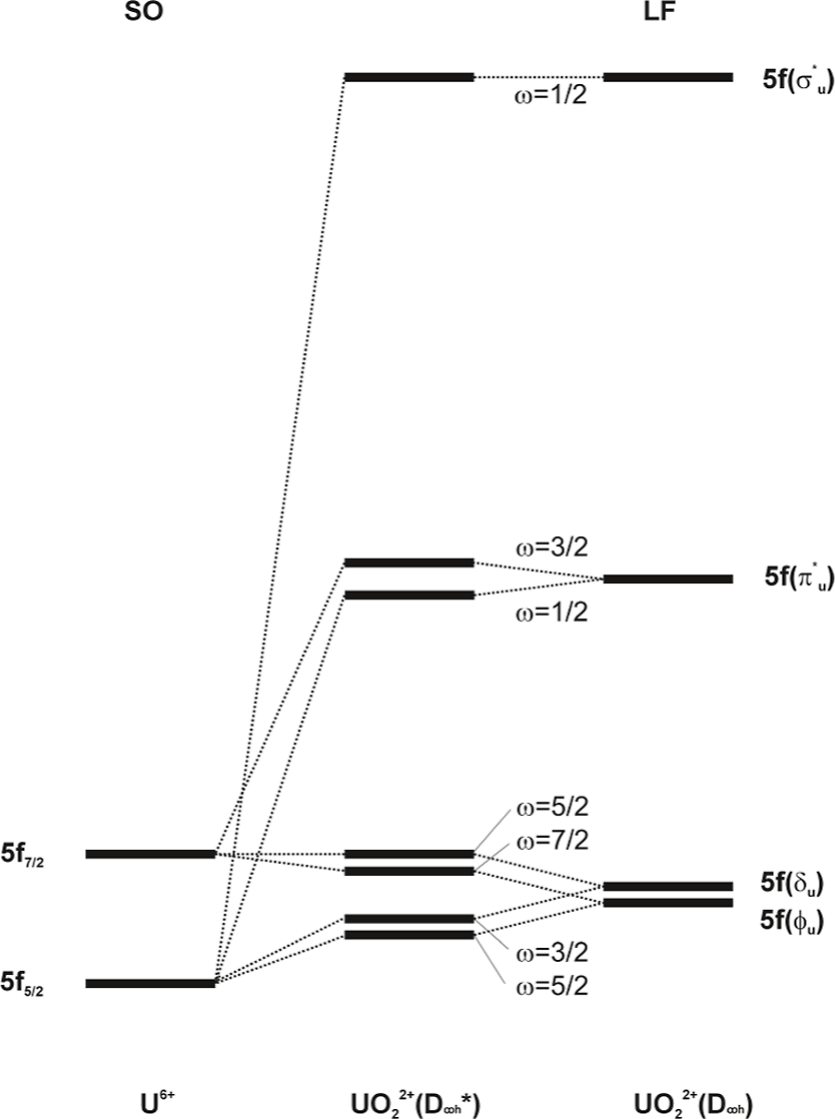
Schematic representation of the spin–orbit (SO) and ligand field (LF) splittings of the dominantly U 5f valence orbitals for uranyl, UO_2_^2+^. The left-hand plot shows the pure atomic SO splitting, and the right-hand plot shows the pure LF splitting into linear symmetry neglecting SO. The correlations are indicated by dotted lines connecting the two extremes of pure SO and pure LF splittings.

The XANES spectral features are observed in many different uranyl systems^4,17–20^ and are assigned to excitations^10,21^ from the 3d core-orbitals into nonbonding 5f ϕ and 5f δ and antibonding 5f π* and 5f σ* open-shell valence orbitals of uranyl (see Figure S1). The charge distribution clearly shows that 5f σ* will be more involved than 5f π* in forming covalent antibonding orbitals with the O anions. If one considers only the 3d and 5f electrons, one can divide the excited states into three groups denoted as |3d^–1^(5f δ/ϕ)^1^⟩, $|3d^{-1}{\left(5f\pi^{*}\right)}^{1}\rangle$ and $|3d^{-1}{\left(5f\sigma^{*}\right)}^{1}\rangle$ although there is some mixing of these configurations. However, once one includes additional many-body effects, this division of the excited states becomes somewhat limited.^22^ Thus, the assignment of an excited state as belonging to one of these groups is somewhat simplistic, precisely because the M_4,5_ excited states cannot, in general, be described as single excitations 3d → 5f but must be represented as multielectron wave functions that mix different core-excited configurations.^10,22,23^

In addition to participating in the nonbonding and antibonding valence orbitals, the U­(5f) orbitals also contribute to the bonding closed-shell uranyl orbitals. Covalent mixing of the empty U­(5f) and U­(6d) orbitals of the isolated U^6+^ cation and the occupied 2p orbitals of the two O^2–^ anions form the bonding closed-shell orbitals with the linear symmetries σ_*u*_, σ_*g*_, π_*u*_, and π_*g*_. The gerade and ungerade bonding orbitals are covalently mixed of the U­(6d) and U­(5f), respectively, with the 2p orbitals of oxygens.

Potential energy curves of the core-excited states give important information related to the U M_5_ edge XANES spectra. In large part, this is because the curvature and equilibrium distance may be quite different for the ground and excited state potential energy curves. In order to identify the importance of these changes in the potential energy curves for the XANES, we have determined these curves for both the ground- and core-excited states, which have dipole-allowed transitions from the ground state. The potential energy curves are determined with multiconfigurational *ab initio* wave functions that include relativistic effects.^10,24–29^ The *ab initio* wave functions are determined with the second-order Douglas–Kroll–Hess (DKH) Hamiltonian,^27,28^ including scalar relativistic effects, and SO splitting is added with perturbation theory.^29^ We calculated the potential energy curves for a relevant range of bond lengths around the equilibrium U–O bond distance of 176 pm^30,31^ deduced from experimental measurements and around the equilibrium bond distance of ≈172 pm obtained from theoretical studies.^32,33^ Both the nonbonding 5f ϕ and 5f δ and antibonding 5f π* and 5f σ* valence open-shell orbitals and the bonding closed-shell orbitals, σ_*u*_, σ_*g*_, π_*u*_, and π_*g*_ of uranyl, will be analyzed in detail for the ground and core-excited states.

In order to determine the covalent character^34^ of the U–O interaction in the ground and core-excited states, mainly due to the interaction of the uranium 5f and 6d orbitals with oxygen, two novel orbital-independent measures are used:^15,16,22,35–37^

(1) The projection of the U­(5f_λ_) and U­(6d_λ_) orbitals of the isolated U^6+^ cation on the bonding closed-shell as well as the valence open-shell orbitals φ_*i*_ of the uranyl molecule, UO_2_^2+^, are denoted as N_p_(*i*, 5f_λ_) and N_p_(*i*, 6d_λ_), respectively. Forming the respective sums over all the 5f_λ_(6d_λ_) orbitals of uranium and all the occupied orbitals φ_*i*_ of uranyl, *N*_p_(5f, |0⟩), and *N*_p_(6d, |0⟩) (see eqs 3 and 4) gives an orbital-independent measure of the 5f and 6d character or occupation of the different states. Specifically, these sums are measures of the properties of the relevant many-electron wave functions.
(2) The size of the closed-shell orbital charge distributions as given by the sum $\sum_{i}{\left\langle z^{2}\right\rangle }_{i}$ , where ${\left\langle z^{2}\right\rangle }_{i}$ is the expectation value of *z* ^2^ for an occupied orbital φ_*i*_, and the origin for the expectation value is the U center. For the calculation of these measures, we used the natural spin orbitals (NSOs).^38^ However, suitable sums over these orbitals are invariant to the choice of orbitals used in the summations.

Both approaches^39^ provide useful measures of how the uranyl electronic structure changes with bond distance.^15,16,22^ It is important that these two measures give consistent views^22^ of the bonding and the chemical interactions. The previous applications of these two methods of analysis to UO_2_^2+^ and other actinide compounds have all involved rigorous 4-component solutions of Dirac Hamiltonians.^40^ Here, we present the first use of these methods with NSOs obtained as variational solutions of the Douglas–Kroll–Hess (DKH) Hamiltonian^27,28^ and SO splitting added with perturbation theory.^29^ If they provide an analysis of the U–O interaction similar to the previous, fully relativistic, analysis, this will permit substantially simpler theoretical treatments of heavy metal systems. Hence, a comparison with data from earlier publications^15,16,22,35–37^ applying these measures to 4-component measures is essential.

The present work reports the potential energy curves of the core-excited states of uranyl, together with properties of the core-excited states of the U M_5_ absorption edge manifold. In our earlier work,^22^ we used rigorous 4-component orbitals obtained as solutions of a Dirac–Fock Hamiltonian where scalar and spin–orbit relativistic effects are included explicitly. However, the orbitals were optimized for an “average of configurations”^33^ for all possible open-shell couplings for an electron distribution with one hole in the U 3d shell and one electron in the nominal U 5f shell. In the present work, the orbitals are optimized separately for the three groups of core-excited states. With this, we obtain different orbitals specific for the different groups of core-excited states and can directly study the changes in the orbitals between the three different groups of core-excited states. These orbitals are obtained as solutions of a 1-component Hamiltonian including scalar relativistic effects^27,28^ and spin–orbit effects are added as a perturbation.^29^ We follow theoretical procedures used in several earlier efforts.^7–10,12,13^ Since we wish to simulate the U M_5_ edge XANES spectra for different U–O distances, the potential energy curves allow us to estimate how much the interaction energy changes for these distances. This is complemented by analyzing the electronic structure at different bond lengths with two novel measures of the covalent character. Hence, we are able to establish a link between X-ray spectroscopy by means of the simulated spectra and chemical bonding.

This article is divided into the following sections: The next section, Section 2, gives a brief overview of the applied theoretical methods and outlines the differences with earlier calculations.^10,22,24–26^ In Section 3, the theoretically determined potential energy curves for the ground (Section 3.1) and the core-excited states (Section 3.2) are shown and discussed. This includes a detailed theoretical analysis of the orbitals and WFs of both the ground- and the core-excited states of the U M_5_ absorption edge manifold of uranyl with dipole allowed transitions from the ground state with variation of the U–O distance. For this we use the projection of the U­(5f) and U­(6d) orbitals of the isolated U^6+^ cation on the orbitals of the uranyl molecule and the size of the charge distribution as given by $\sum_{i}{\left\langle z^{2}\right\rangle }_{i}$ . A particular focus is the variation with the bond length and how they indicate changes in the covalent character with distance. In Section 3.3, we present the simulated XANES spectra for different U–O bond length, and we compare our predicted spectra with data derived from experimental measurements.^16,22^ In Section 4, we combine our spectroscopic results with the analysis of the wave function and establish a link between X-ray spectroscopy and chemical bonding of uranyl. The paper closes with Section 5, where conclusions reached by the presented data about the chemistry of the ground and relevant core-excited states of UO_2_^2+^ are reviewed

## 2 Methods

The simple description of the U M_5_ absorption edge as one-electron excitation from the 3d_5/2_ core orbitals of uranium to the empty 5f_5/2,7/2_ valence orbitals of uranium is oversimplified and incomplete. In order to correctly describe the excitation energies and the wave function properties, a more general many-electron treatment is needed. This treatment is best described in terms of configurations where the active electrons are distributed with different occupations of active orbitals. Indeed, this is precisely the way the wave function is constructed in a configuration interaction, CI, methodology.^41,42^ When the many-electron treatment includes only distribution of the active valence shell electrons over the open-shell, 3d_5/2_ and 5f_5/2,7/2_ orbitals, this is described as a treatment of static many-electron effects. When the many electron treatment also includes excitations from nominally closed-shell orbitals into the open-shell space this is described as a treatment that includes dynamic electron effects.^43^ The static correlation accounts for the angular momentum coupling of the open-shell electrons, which is necessary in order to have wave functions with the correct total symmetry. The dynamic correlation effects are needed to obtain more accurate energy differences for the excited states and to be able to describe the satellites as well as the main features in the X-ray adsorption spectra. In particular, the angular momentum coupling of the core, 3d_5/2_, open-shell, and the valence, dominantly U­(5f) open-shell electrons, must be taken into account.^22^ The multiplets arising from the simultaneous presence of these two sets of open shells in the core-excited states require multiconfigurational methods to describe the electronic structure adequately.

Scalar relativistic effects are considered with the second-order Douglas–Kroll–Hess (DKH) Hamiltonian.^27,28^ Spin–orbit relativistic effects are included by perturbation theory using a mean-field SO operator^29^ based on the effective one-electron Fock-type spin–orbit Hamiltonian, as suggested by Hess et al.^27,28^ Restricted active space self-consistent field (RASSCF)^43–46^ calculations are used to treat the static correlation when describing open-shell configurations with one hole in the 3d core electron shell and a 5f valence orbital occupied. Dynamic correlation is recovered using second-order perturbation theory (RASPT2)^47,48^ method. The choice of the active spaces in the HR-XANES is the same as in our previous calculations^10^ (see Section S1.2 in the Supporting Information).

In this work, we are especially interested in the difference of the U–O bonding closed-shell orbitals, σ_*u*_, σ_*g*_, π_*u*_, and π_*g*_, between the three sets of core-excited states in the uranyl UO_2_^2+^ U M_5_ absorption edge and how they differ from the corresponding orbitals of the ground state. Therefore, we did separate calculations for each of the three different sets of core-excited states by restricting the state-averaging to one set of core-excited states at a time. With this approach, the orbitals are optimized individually for the three different sets, and the differences between the orbitals can be analyzed.

In our recent work,^22^ we used two theoretical models: Open-Shell Active (OSA) and Closed-Shell Active (OCSA). Here, we consider the bonding closed-shell orbitals σ_*u*_, σ_*g*_, π_*u*_, and π_*g*_ inactive and doubly occupied in all configurations, similar to the OSA model. In the current work, improved accuracy is reached by the restriction of the state-averaging, as outlined above.

The calculations of the projections *N*_p_(5f, |0⟩) (eq 3) and *N*_p_(6d, |0⟩) (eq 4) of atomic U­(5f_λ_) and U­(6d_λ_) fragment orbitals of the isolated U­(VI) cation on the molecular orbital φ_*i*_ of UO_2_^2+^ using the projection operator ${\mathrm{P̂}}_{i,U}^{AO}$ (see eq 1 in ref 37) are outlined in detail in ref 22,37 ; here, we give only a brief outline. The projections, *N*_p_

1 $$N_{p}\left(i,5f_{\lambda }\right)=|\langle U\left(5f_{\lambda }\right)|\varphi_{i}\rangle {|}^{2}$$

2 $$N_{p}\left(i,6d_{\lambda }\right)=|\langle U\left(6d_{\lambda }\right)|\varphi_{i}\rangle {|}^{2}$$

are simply the squares of the overlap integrals between the isolated orbitals of the U­(VI) cation and the *i*th uranyl orbital. These projections are properly viewed as cation occupations in the compound orbitals. Thus, *N*_p_(*i*, 5f_λ_) is the 5f_λ_ occupation of the molecular orbital φ_*i*_. If *N*_p_(*i*, 5f_λ_) = 1, φ_*i*_ is a pure 5f_λ_ orbital, and if *N*_p_(*i*, 5f_λ_) = 0, φ_*i*_ has no 5f_λ_ character. Intermediate values indicate a covalent interaction in φ_*i*_ with 5f_λ_ participation with similar comments for the 6d occupations. In general, different linear symmetries divide the occupations into σ, π, etc. characters. The total 5f and 6d character or occupation of the ground state configuration is given by the sums

3 $$N_{p}\left(5f,|0\rangle \right)=\sum_{\lambda }\sum_{i\in closed}N_{p}\left(i,5f_{\lambda }\right),\left(\lambda =1-7\right)$$

and

4 $$N_{p}\left(6d,|0\rangle \right)=\sum_{\lambda }\sum_{i\in closed}N_{p}\left(i,6d_{\lambda }\right),\left(\lambda =1-5\right)$$

(the designation for the core-excited states is accordingly *N*_p_(5f, |3d^–1^(5f δ/ϕ)^1^⟩), ···). Note that since these are nonrelativistic orbitals, we do not need to include a sum over spin since the spin orbitals are simple products of the space and the spin, α or β. A word of caution is in order for these projections that overlap with the diffuse O­(2p) orbitals to introduce artifacts in the nominal U occupations. This is especially important for the occupation of the U­(6d) orbitals. These possible limitations are discussed in more detail below, and a possible correction is used to avoid the artifacts. The 5f_λ_ and 6d_λ_ spin orbitals of the isolated U­(VI) cation can be rotated to have linear symmetry of UO_2_^2+^ and projections on the uranyl orbitals are only nonzero if they have the appropriate symmetry of the atomic orbitals. Further, the 5f projections are nonzero only for UO_2_^2+^ which have the ungerade symmetry of the 5f orbitals; similarly the 6d projections are nonzero only for UO_2_^2+^ orbitals which have gerade symmetry. When summing over all 5f_λ_ or 6d_λ_ orbitals of the isolated U­(+VI) cation, and all the closed-shell orbitals φ_*i*∈closed_ of the ground state or the core-excited states, we obtain direct measure of the 5f or 6d character of the closed-shell orbitals of these states. Since *N*_p_(5f, |0⟩), *N*_p_(5f, |3d^–1^(5f δ/ϕ)^1^⟩), etc. are orbital invariant, they do not depend on orbital transformations like localization. However, it is important to recall that the summation over the closed-shell uranyl orbitals is possible because these orbitals are all fully occupied in all configurations included in the wave function. The variation of the sums in eqs 3 and 4 gives clear information about the variation of the total 5f or 6d closed-shell bonding character in the states as the U–O distance is varied. Additionally, they provide a good measure of the extent to which the electrons of the two oxygen anions fill the 5f and 6d orbitals of uranium for different bond lengths. Again, it is important to note that a correction as described below (see eqs 6 and 7) is needed, especially for the diffuse 6d orbital projections. This sum can be used to estimate covalent character in the wave functions of the different many-body states.

For the ground state where there are no open-shell electrons, the closed shells are the only way that the wave functions can have either 5f or 6d character; this is not the case for the 5f ungerade character of the excited states. For the core-excited states, e.g., the |3d^–1^(5f δ/ϕ)^1^⟩ states, a different summation must be used to estimate the occupations in the open shells; see ref 22. We sum over open-shell valence orbitals φ_*i*_ and all 5f_λ_ or 6d_λ_ orbitals of the isolated U­(+VI) cation

5 $$N_{p}^{val}\left(5f,|3d^{-1}{\left(5f\delta /\phi \right)}^{1}\rangle \right)=\sum_{\lambda }\sum_{i\in valence}N_{p}\left(i,5f_{\lambda }\right),\left(\lambda =1-7\right)$$

which gives the 5f character of these open-shell valence orbitals. As for previous summations, it is not necessary to include spin for these summations since the orbitals are nonrelativistic.

Since the spatial extent of the Uranium 5f and 6d frontier orbitals are quite large, we calculated additionally the projection of the orbitals of the isolated U­(VI) cation on the orbitals of one isolated O­(–II) anion, N_p_(⟨U^6+^|O^2–^⟩)­(*j*, 5f_λ_) and N_p_(⟨U^6+^|O^2–^⟩)­(*j*, 6d_λ_), and corrected the initial *N*_p_(5f, |0⟩) (see eq 3) and *N*_p_(6d, |0⟩) (see eq 4) for this artificial contribution by summing over all occupied atomic orbitals $\varphi_{j,O^{2-}}$ of the oxygen anion

$$\begin{array}{c} N_{p,corr}\left(5f,|0\rangle \right)=N_{p}\left(5f,|0\rangle \right)\\ -2\cdot \underset{\lambda }{\sum }\underset{j\in occupied\left(O^{2-}\right)}{\sum }N_{p}\left(\langle U^{6+}|O^{2-}\rangle \right)\left(j,5f_{\lambda }\right),\left(\lambda =1-7\right) \end{array}$$ 6

$$\begin{array}{c} N_{p,corr}\left(6d,|0\rangle \right)=N_{p}\left(6d,|0\rangle \right)\\ -2\cdot \underset{\lambda }{\sum }\underset{j\in occupied\left(O^{2-}\right)}{\sum }N_{p}\left(\langle U^{6+}|O^{2-}\rangle \right)\left(j,6d_{\lambda }\right),\left(\lambda =1-5\right) \end{array}$$ 7

The corrected values *N*_p,corr_(5f, |0⟩) and *N*_p,corr_(6d, |0⟩) give a better estimate for the covalent character.

The calculation of the ⟨*z* ^2^⟩ is used as a measure for the size of the charge distributions.^22^ The expectation value of ⟨*z* ^2^⟩ for the total wave function is the sum over the orbital expectation values $\sum_{i\in occupied}{\left\langle z^{2}\right\rangle }_{i}$ for all orbitals. We study the variation of this measure with the bond length. The 5f and 6d occupations are distinguished by separating the sums over the gerade and ungerade uranyl orbitals. The sums, $\sum_{i\in g,u}{\left\langle z^{2}\right\rangle }_{i}$ , increase significantly as the bond length increases. This is to be expected since a significant fraction of the 10 electrons are formally associated with the O^2–^ anion will move with the anions, and as the anions move away from the origin, the sum of $\sum_{i\in g,u}{\left\langle z^{2}\right\rangle }_{i}$ will be increased. Comparing the sums $\sum_{i\in g,u}{\left\langle z^{2}\right\rangle }_{i}$ between the ground and core-excited states indicates changes in the spatial extent of the gerade and ungerade orbitals. An important further information is contained in the slopes of $\sum_{i\in g,u}{\left\langle z^{2}\right\rangle }_{i}$ . As the electrons move with the anion, larger covalency is an attractive interaction toward cation U^6+^ and causes the ⟨*z* ^2^⟩ to increase less rapidly than it would if one simply moved an anion with a nominal charge of −2. Therefore, the slope of $\sum_{i\in g,u}{\left\langle z^{2}\right\rangle }_{i}$ is a measure for covalency, and smaller slopes correspond to a more covalent interaction. For the core excited states, the $\sum_{i\in g,u}{\left\langle z^{2}\right\rangle }_{i}$ gives, in addition, a very good measure of the separate changes in the covalent character of g and u orbitals. specifically, U­(6d) and U­(5f).

OpenMolcas^49^ was used for the calculations. MS-RASPT2 ^47^ calculations were performed with the ionization potential electron affinity parameter (IPEA) of 0 *E*_h_ and an imaginary shift of 0.2 *E*_h_. The nominal symmetry used for the calculations was *C*_2*h*_ but all the orbitals and wave functions have the correct *D*_2*h*_ or *D*_2h_^*^ (double group) symmetry. The molecular axis is oriented in the *z* direction. Relativistic atomic natural orbital basis sets ANO-RCC-VTZ^50,51^ were used for the calculations. The basis sets have been contracted using the DKH Hamiltonian and optimized to be used in calculations where scalar relativistic effects are included.

## 3 Results

The potential energy curves of the ground as well as the core-excited states of uranyl UO_2_^2+^ belonging to the U M_5_ absorption edge manifolds are shown in Figure 2. Only curves of core-excited states with dipole-allowed transitions from the ground state are considered (Table 1). The potential energy curves of the states with the highest oscillator strengths for each group of core-excited states are shown in Figure 2a. The corresponding spectroscopic parameters are listed in Table 2. The potential energy curves of the core-excited states corresponding to excitations into the |3d^–1^(5f δ/ϕ)^1^⟩, $|3d^{-1}{\left(5f\pi^{*}\right)}^{1}\rangle$ and $|3d^{-1}{\left(5f\sigma^{*}\right)}^{1}\rangle$ groups are shown in red (Figure 2b), green (Figure 2c), and blue (Figure 2d), respectively. There are $6|0_{u}^{+}\rangle$ and 11 2-fold degenerate |1_u_⟩ core-excited states, and they are enumerated, in energetic ascending order, with Roman numerals: ${|0_{u}^{+}\rangle }_{I},...,{|0_{u}^{+}\rangle }_{VI}$ and ${|1_{u}\rangle }_{I},...,{|1_{u}\rangle }_{XI}$ in Tables 1 and 2 as well as in Figure 2. These states are most important for the interpretation of the U M_5_ edge XANES. The potential energy curves were determined around the reported experimental assumed equilibrium structure of 176 pm.^30,31^

**Figure 2:**
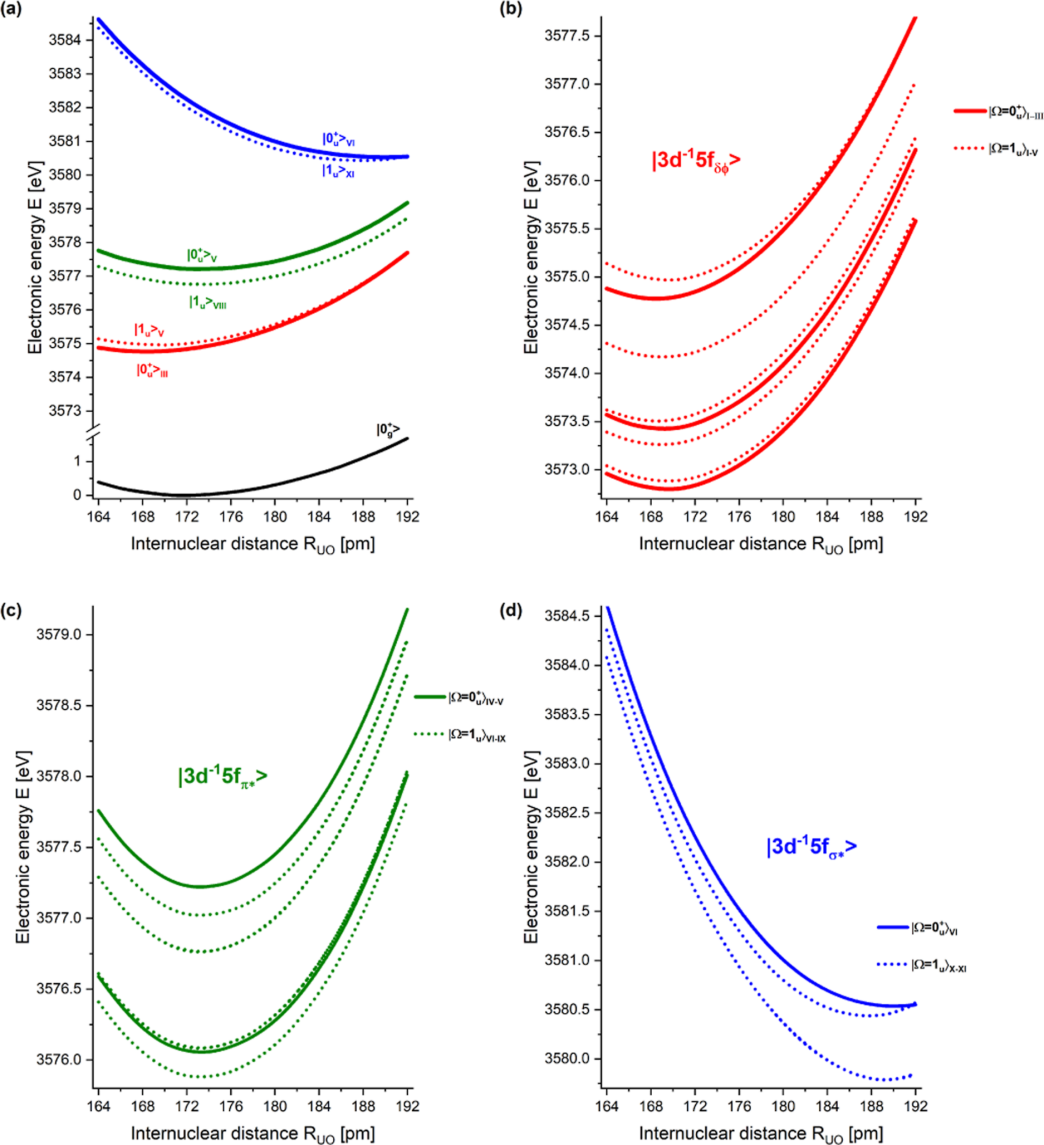
Potential energy curves, with energy in eV and distances in pm, of the |3d^–1^(5f δ/ϕ)^1^⟩, $|3d^{-1}{\left(5f\pi^{*}\right)}^{1}\rangle$ and $|3d^{-1}{\left(5f\sigma^{*}\right)}^{1}\rangle$ core-excited states of uranyl UO_2_^2+^ belonging to the U M_5_ manifold. The symmetry to consider is Ω. Only curves of states with dipole-allowed transitions (ΔΩ = 0, ±1) from the ground state are shown. The potential energy curves of the core-excited states corresponding to excitations into the |3d^–1^(5f δ/ϕ)^1^⟩, the $|3d^{-1}{\left(5f\pi^{*}\right)}^{1}\rangle$ and the $|3d^{-1}{\left(5f\sigma^{*}\right)}^{1}\rangle$ groups are shown in red, green, and blue, respectively. Potential energy curves of the states with Ω = 0 are shown with solid lines and states with Ω = 1 with dotted lines, respectively. (a) Potential energy curves of states with the highest oscillator strengths for excitations from the ground state for each group of core-excited states. (b) Potential energy curves of states belonging to the |3d^–1^(5f δ/ϕ)^1^⟩ group. (c) Potential energy curves of states belonging to the $|3d^{-1}{\left(5f\pi^{*}\right)}^{1}\rangle$ group. (d) Potential energy curves of states belonging to the $|3d^{-1}{\left(5f\sigma^{*}\right)}^{1}\rangle$ group.

**Table 1:** Symmetries (*Sym*), Excitation Energies (*E* in [eV]), Oscillator Strengths (f), and Occupation Numbers of the 3d Core-Orbitals (*n*_3d_) and of the 5f Valence Orbitals (*n*_5f_) for the Core-Excited States with an Oscillator Strength *f* ≥*f*_max_/100 at *R* = 176 pm are given^a^

U M_5_ edge										
				occupation numbers						
		*E*	*f*	*n*_3d_ core-hole			*n*_5f_ valence orbital			
Sym	i	[eV]	*f*/*f*_max_	3d δ	3d π	3d σ	5f ϕ	5f δ	5f π*	5f σ*
3d → 5f δϕ										
${|0_{u}^{+}\rangle }_{I}$	1	3572.9	<0.01	–	–	––	–	–	–	
${|1_{u}\rangle }_{I}$	2	3573.0	<0.01	–	–	––	–	–	–	
${|1_{u}\rangle }_{II}$	3	3573.5	<0.01	–	–	––	–	–	–	
${|0_{u}^{+}\rangle }_{II}$	4	3573.6	0.02	–0.40	–0.60	0.00	0.24	0.72	0.03	0.00
${|1_{u}\rangle }_{III}$	5	3573.7	<0.01	–	–	––	–	–	–	–
${|1_{u}\rangle }_{IV}$	6	3574.3	<0.01	–	–	––	–	–	–	–
${|0_{u}^{+}\rangle }_{III}$	7	**3575.0**	**0.27**	**–0.94**	**–0.04**	**–0.02**	**0.03**	**0.90**	**0.06**	**0.00**
${|1_{u}\rangle }_{V}$	8	**3575.1**	**0.96**	**–0.54**	**–0.36**	**–0.10**	**0.57**	**0.30**	**0.12**	**0.00**
3d → 5f π*										
${|1_{u}\rangle }_{VI}$	9	3575.8	0.02	–0.06	–0.56	–0.39	0.00	0.02	0.97	0.01
${|0_{u}^{+}\rangle }_{IV}$	10	3576.0	0.19	–0.04	–0.40	–0.56	0.00	0.02	0.97	0.01
${|1_{u}\rangle }_{VII}$	11	3576.0	0.27	–0.10	–0.61	–0.29	0.03	0.01	0.93	0.03
${|1_{u}\rangle }_{VIII}$	12	**3576.7**	**0.52**	**–0.05**	**–0.38**	**–0.57**	**0.02**	**0.06**	**0.92**	**0.00**
${|1_{u}\rangle }_{IX}$	13	3577.0	0.35	–0.02	–0.96	–0.00	0.02	0.02	0.97	0.00
${|0_{u}^{+}\rangle }_{V}$	14	**3577.2**	**1.00**	**–0.22**	**–0.76**	**–0.03**	**0.02**	**0.02**	**0.97**	**0.00**
3d → 5f σ*										
${|1_{u}\rangle }_{X}$	15	3580.8	0.01	–0.02	–0.48	–0.50	0.00	0.00	0.00	0.98
${|1_{u}\rangle }_{XI}$	16	**3581.2**	**0.26**	**–0.08**	**–0.20**	**–0.72**	**0.00**	**0.00**	**0.02**	**0.98**
${|0_{u}^{+}\rangle }_{VI}$	17	**3581.4**	**0.52**	**–0.01**	**–0.40**	**–0.59**	**0.00**	**0.00**	**0.04**	**0.96**

a The occupation numbers *n*_3d_ refer to an occupation of 2 + *n*_3d_ for the 3d σ and 4 + *n*_3d_ for the 3d π/δ orbitals. The states ( ${|0_{u}^{+}\rangle }_{III}$ , ${|0_{u}^{+}\rangle }_{V}$ , ${|0_{u}^{+}\rangle }_{VI}$ , ${|1_{u}\rangle }_{V}$ , ${|1_{u}\rangle }_{VIII}$ and ${|1_{u}\rangle }_{XI}$ ) are shown in Figure 2a and their spectroscopic parameters are reported in Table 2.

**Table 2:** Spectroscopic Parameters of the Ground and Selected Core-Excited States with the Highest Oscillator Strengths for Each Group of Core-Excited States in Energetic Ascending Order (See Figure 2a)^a^

State	i	*R*_*i*_ [pm]	Δ*R*_*i*_ = *R*_*i*_ – *R*_0_ [pm]	*E*_*i*_ [eV]	Δ*E*_*i*_ [eV]	ω_*e*,*i*_ [cm^–1^]	Δω_*e*,*i*_ = ω_*e*,*i*_ – ω_*e*,0_ [cm^–1^]	occupied open-shell valence orbital
Ground State								
|0_g_^+^⟩	-	172.0	–	0.0	–	997	–	–
Core-Excited States								
${|0_{u}^{+}\rangle }_{III}$	7	168.4	–3.2	3574.8	0.0	982	–15	5f_δ_
${|1_{u}\rangle }_{V}$	8	169.4	–2.2	3575.0	0.2	1012	+17	5f_δ,ϕ_
${|1_{u}\rangle }_{VIII}$	12	173.4	+1.4	3576.8	2.0	1008	+11	5f_π*_
${|0_{u}^{+}\rangle }_{V}$	14	173.5	+1.5	3577.2	2.4	993	–4	5f_π*_
${|1_{u}\rangle }_{XI}$	16	187.5	+15.5	3580.4	5.6	1067	+70	5f_σ*_
${|0_{u}^{+}\rangle }_{VI}$	17	190.0	+18.0	3580.5	5.7	859	–138	5f_σ*_

a Equilibrium structures *R*_*i*_ and the corresponding electronic energies *E*_*i*_(*R*_*i*_) are provided. Additionally, we provide Δ*E* = *E*_*i*_ – *E*_7_ and the harmonic vibrational frequency ω_*e*,*i*_ (the labels i refer to Table 1).

### 3.1 Potential Energy Curve of the Ground State |0_*g*_^+^⟩

The symmetry to consider is Ω and the ground state has a total symmetry |0_*g*_^+^⟩. The equilibrium U–O bond distance for the ground state is *R*_UO_ ≈ 172 pm, and the harmonic vibrational frequency ω_*e*,*i*_ for the ground state is 997 cm^–1^ (see Figure 2a and Table 2). The equilibrium bond distance and harmonic vibrational frequencies are determined by fitting a quadratic function to the points around the equilibrium structure. These results are quite close to previously reported theoretical results of 171.5 pm and 974 cm^–1^ by Pierloot and van Besien^32^ and de Jong et al.^33^ It is interesting to note that the theoretical equilibrium distance of *R*_UO_ ≈ 172 pm is quite close to the assumed distance of ≈176 pm, as well, which has been used in the analysis of experiments.^21,30,31^

The reasons for the relatively minor differences between our results and the previous results^32,33^ may involve different choices in the polynomial fits to the theoretical potential curve and to the fact that we are using a somewhat different active space in our *ab initio* many-body calculations.

#### 3.1.1 Characterization of the Ground State |0_*g*_^+^⟩ of Uranyl for Varying Bond Lengths

The electronic configuration of the uranyl ground state |0_*g*_^+^⟩ has a U^6+^ occupation of 6s^2^6p^6^5f^0^6d^0^; however, the higher lying closed-shell uranyl orbitals do have modest to large amounts of bonding covalent character. The covalent character does not change significantly in the considered range of bond distances around the equilibrium distance, as will be shown below.

The bonding closed-shell orbitals, σ_*u*_, σ_*g*_, π_*u*_, and π_*g*_, are formed by covalent mixing of the Uranium 5f (σ_*u*_ and π_*u*_) and 6d (σ_*g*_ and π_*g*_) and oxygen orbitals. The nominal charges of the atoms forming uranyls are U­(+VI) and O­(–II). Since there is considerable covalency in uranyl, the actual effective charges are significantly different from these nominal charges. The projections *N*_p,corr_(5f, |0⟩) and *N*_p,corr_(6d, |0⟩) provide a good measure to which extent the electrons of the two O­(–II) anions fill the 5f and 6d orbitals of uranium for different U–O bond lengths when forming these bonding orbitals.

The corrected values, *N*_p,corr_(5f, |0⟩) (eq 6) and *N*_p,corr_(6d, |0⟩) (eq 7), together with the uncorrected values, *N*_p_(5f, |0⟩) (eq 3) and *N*_p_(6d, |0⟩) (eq 4), are shown in Figure 3. The uncorrected results shown in Figure 3 agree with the results presented previously for the ground state (see Figure 9 in ref 22), which shows that the approximations in our present approach are satisfactory.

**Figure 3:**
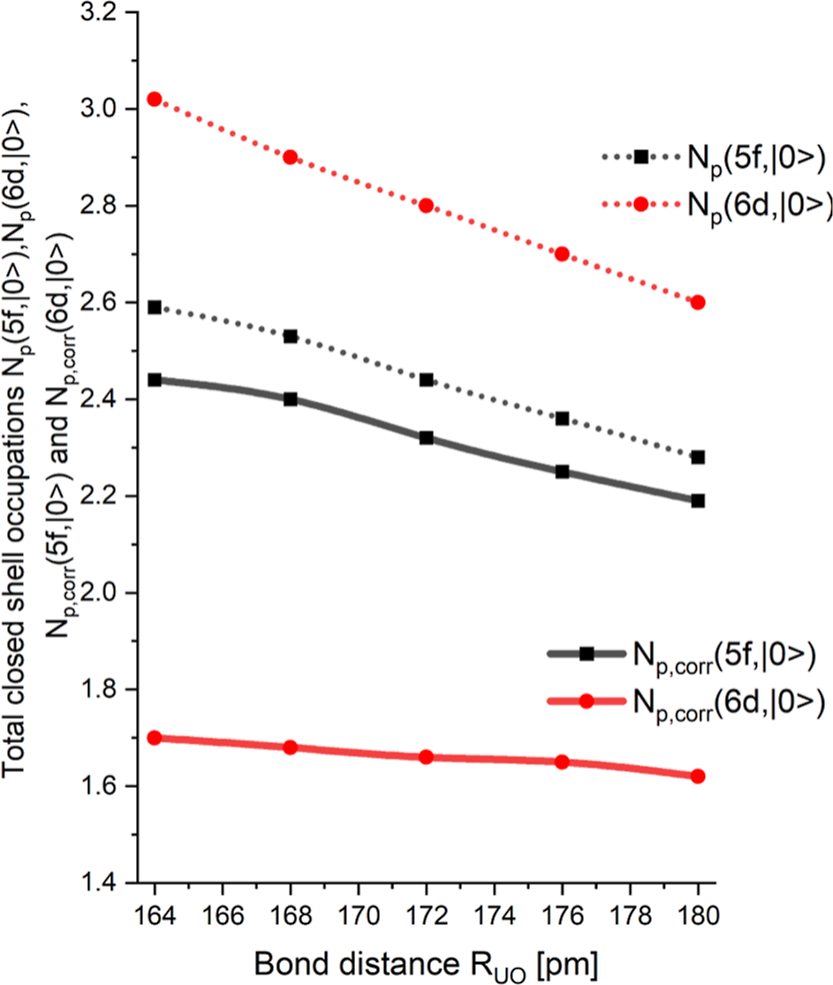
Projections *N*_p,corr_(5f, |0⟩) and *N*_p,corr_(6d, |0⟩) of the ground state of uranyl as a function of the bond distance around the equilibrium structure together with their uncorrected counter parts *N*_p_(5f, |0⟩) and *N*_p_(6d, |0⟩). The uncorrected data is shown as dotted, and the corrected as solid lines.

The differences between *N*_p,corr_(5f, |0⟩) and *N*_p_(5f, |0⟩) are quite small (≈5% of the uncorrected values), but the changes between *N*_p,corr_(6d, |0⟩) and *N*_p_(6d, |0⟩) for the 6d occupations are substantial (≈40% of the uncorrected values). The reason for this is the very large spatial extension of the 6d orbitals compared to the 5f orbitals. Therefore, the corrections have to be included to arrive at reliable values of the covalent character and to obtain the correct ordering of the 5f and 6d occupation in the closed-shell bonding orbitals. The following discussion refers only to the corrected values *N*_p,corr_(5f, |0⟩) and *N*_p,corr_(6d, |0⟩).

*N*_p,corr_(5f, |0⟩) and *N*_p,corr_(6d, |0⟩) in Figure 3 indicate that there is substantial 5f and 6d character in the ground state of uranyl, but there is less but still significant 6d character. At *R* = 172 pm we have a 5f occupation of 2.32 and a 6d occupation of 1.66. These assignments of 6d and 5f charges would suggest that the O “anions” are almost neutral. Clearly this shows limitations to the quantitative view of the projections of 5f and, especially, 6d character. In particular, the 6d projections are highly suspect since the apparent 6d character may simply reflect the fact that the O^2–^ charge distributions are not spherically but substantially polarized by the presence of the U­(VI) cation. However, the result indicates more 5f than 6d character in the closed-shell bonding orbitals and a monotonic increase with shorter U–O bond length. This result is in very good agreement with the data reported in our earlier work.^22^ Here, we show additionally that the applied corrections, *N*_p,corr_(5f, |0⟩) and *N*_p,corr_(6d, |0⟩), are essential to arrive at the true values of the 5f and 6d character in the bonding closed-shell orbitals.

When summing over the occupied molecular orbitals φ_*i*_ of uranyl in eqs 3 and 4, it is interesting to identify which molecular orbitals of uranyl have significant 5f and 6d occupation. We use two different sets of orbitals to study the 5f and 6d occupation, canonical and localized orbitals.^52^ With both sets, we find that the uranyl bond is formed by the 2*p* atomic orbitals of the oxygens and the 5f and 6d orbitals of Uranium.

The U–O distance in uranyl depends on the environment of the uranyl; for a large class of uranyl compounds, the U–O bond distances range from 176 to 181 pm.^4^ Our data shows that in this range the 5f occupation of the ground state changes only by ≈2.7%. If this is translated into an increase of covalency, we can directly quantify to which extent covalency is increased when the bond distance is shortened from 181 to 176 pm.

The second measure giving information about the covalent character of the U–O bond is the spatial extent of the orbitals by means of the spatial extent of the closed-shell charge density $\sum_{i}{\left\langle z^{2}\right\rangle }_{i}$ of the bonding closed-shell orbitals φ_*i*_. If the orbital is primarily located at U, the values of $\sum_{i}{\left\langle z^{2}\right\rangle }_{i}$ should be rather small, whereas for orbitals located at the oxygens, it should be about, and possibly slightly larger than, the uranyl bond distance *R*.

Figure 4a,b shows the separate sums, $\sum_{u,g}{\left\langle z^{2}\right\rangle }_{i}$ , over the (a) gerade and (b) ungerade orbitals for the ground state as well as the core-excited states. Here, we discuss only the curves for the ground state, which are shown in black. The information contained in the curves for the core-excited states will be discussed in Section 3.2.1. The sums over the gerade states provide information about the interaction of the anions with the U­(6d) orbitals and the sum over ungerade states with the U­(5f) orbitals.

**Figure 4:**
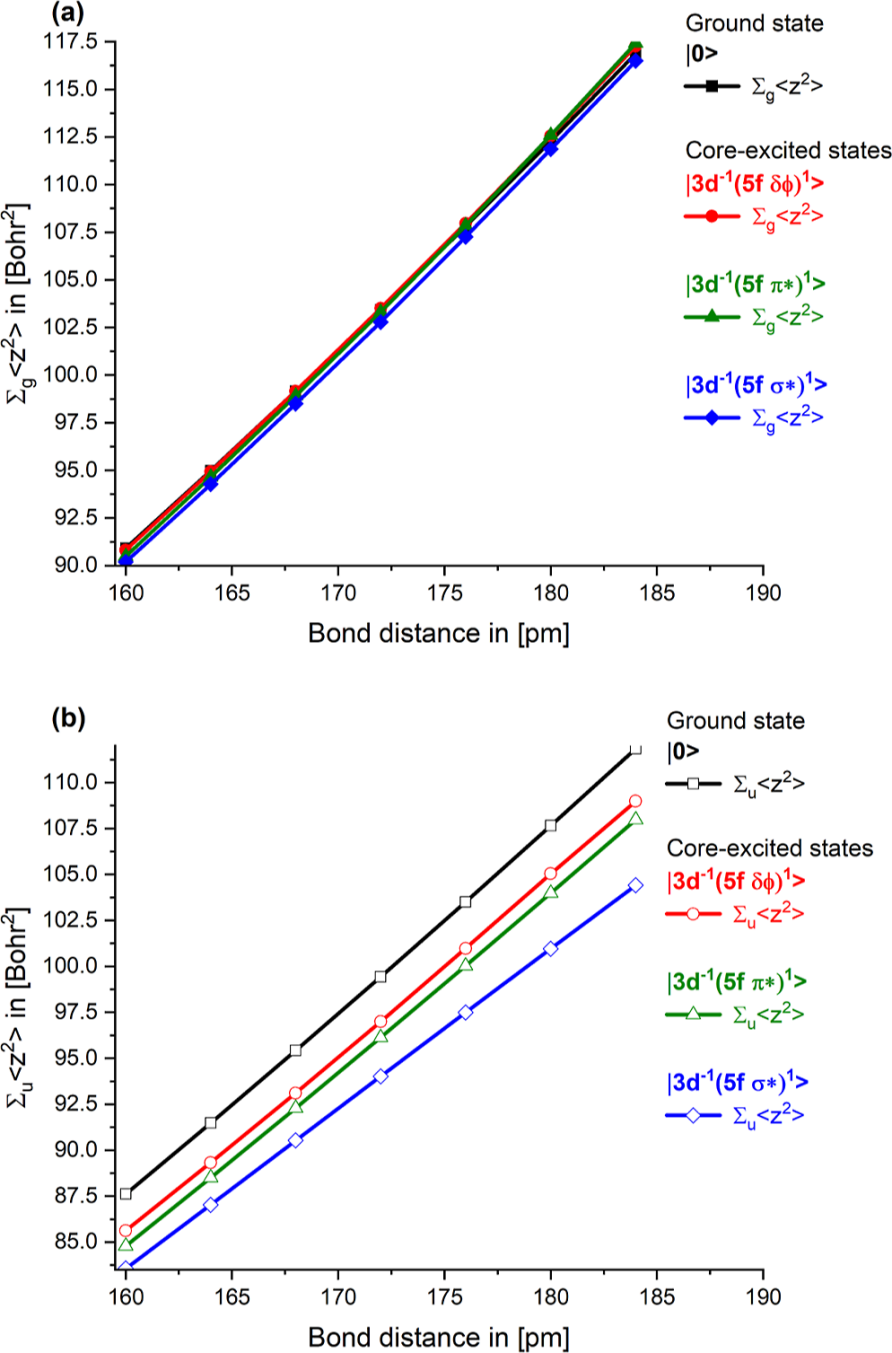
Dependence of the sums $\sum_{u,g}{\left\langle z^{2}\right\rangle }_{i}$ on the bond length *R*_UO_ for the ground state and the core-excites states. (a) Sum over gerade orbitals is shown with filled squares, whereas the sum over (b) ungerade orbitals is shown with open squares. The curves for the ground state are shown in black. The curves are for the |3d^–1^(5f δ/ϕ)^1^⟩ core-excited state in red, for the $|3d^{-1}{\left(5f\pi^{*}\right)}^{1}\rangle$ states in green, and for the $|3d^{-1}{\left(5f\sigma^{*}\right)}^{1}\rangle$ states in blue.

Both sums increase significantly as the bond length increases. The slope of the sum over ungerade orbitals is smaller compared to the slope of the sum over gerade orbitals, indicating that there is more covalent interaction in the ungerade orbitals due to the interaction of the U­(5f) orbitals with the oxygen anions compared to the covalency in the gerade states due to the covalent mixing with the U­(6d) orbitals.

This consideration using the sums, $\sum_{u,g}{\left\langle z^{2}\right\rangle }_{i}$ , confirms the result using the projections *N*_p,corr_(5f, |0⟩) and *N*_p,corr_(6d, |0⟩). For *N*_p,corr_(5f, |0⟩) and *N*_p,corr_(6d, |0⟩), we found a larger 5f character compared to the 6d character. Hence, the results of both measures for the covalency provide consistent results, and we conclude that covalency is monotonically increasing with shorter bond length for the UO_2_^2+^ ground state and that we have more 5f than 6d character in the closed-shell bonding orbitals. Additionally, the results agree with our earlier findings.^22^

### 3.2 Potential Energy Curve of the Core-Excited States

For the U M_5_ absorption edge, there are 84 states, including spin–orbit (SO) coupling, resulting from single electron excitations from the ground state of uranyl to core-excited states with configuration 3d_5/2_ → 5f_5/2,7/2_. This is obtained as the product of 6 3d_5/2_ holes with an electron in one of the 14 5f spin orbitals. However, many of these states have the same energy, and there are fewer distinct multiplets. The ground state has a total symmetry 0_*g*_^+^, and the selection rules for dipole-allowed transitions are ΔΩ = 0, ±1, *g* ↔ *u* and + ↔ +. There are $6|0_{u}^{+}\rangle$ and 11 2-fold degenerate |1_*u*_⟩ core-excited states with dipole allowed excitations in the U M_5_ absorption edge manifold of uranyl. They are enumerated in energetic ascending order with Roman numerals in Table 1. Please be aware that the energies in Table 1 and Table 2 differ slightly because they are at different U–O distances.

Figure 2 shows the potential energy curves of the |0_*u*_^+^⟩ and |1_*u*_⟩ core-excited states with dipole-allowed transitions from the ground state (see Table 1). Potential energy curves of the ground and selected core-excited states with the highest oscillator strengths for each group of core-excited states (Table 1) are presented in Figure 2a. Figure 2b–d shows the three distinct branches which correspond to the three groups of core-excited states |3d^–1^(5f δ/ϕ)^1^⟩, $|3d^{-1}{\left(5f\pi^{*}\right)}^{1}\rangle$ , and $|3d^{-1}{\left(5f\sigma^{*}\right)}^{1}\rangle$ . These states are the only states relevant to understanding the U M_5_ edge XANES spectra.

The shapes of these three branches are quite distinct from each other. Table 2 summarizes the spectroscopic parameters for the core-excited states with the highest oscillator strengths for each group of core-excited states. The $|3d^{-1}{\left(5f\sigma^{*}\right)}^{1}\rangle$ branches with the highest energy (shown in blue in Figure 2d) display a nonbonding shape with shallow minima at ≈187.5–190 pm. The harmonic vibrational frequencies for these states differ also significantly from the result for the ground state. The other two branches, $|3d^{-1}{\left(5f\pi^{*}\right)}^{1}\rangle$ and |3d^–1^(5f δ/ϕ)^1^⟩, shown in green and red, respectively, in Figure 2b,c, have similar shapes as the ground state and minima around the equilibrium distance of the ground state.

The reason for these very different shapes of the three branches is the occupation of the antibonding 5f σ* orbital (see Figure S1) in the $|3d^{-1}{\left(5f\sigma^{*}\right)}^{1}\rangle$ branch. Therefore, these core-excited states have a substantial repulsive shape. This is different for the other two branches. In the $|3d^{-1}{\left(5f\pi^{*}\right)}^{1}\rangle$ branch the antibonding 5f π* is occupied, and in the |3d^–1^(5f δ/ϕ)^1^⟩ branch the nonbonding 5f δ/ϕ orbitals.

#### 3.2.1 Characterization of the Core-Excited States of the Uranyl UO_2_^2+^ U M_5_ Absorption Edge Manifold for Varying Bond Lengths

The main focus is on the variation of the electronic structure with the bond length for the various core-excited states. Relative to the ground state, there are changes induced in the electronic structure by the core-hole in the 3d shell and the simultaneous population of one 5f valence open-shell orbital.

When characterizing the core excited states of the U M_5_ absorption edge in addition to the bonding closed-shell orbitals, the valence open-shell orbitals have to be considered as well. We focus on the variation of both sets of orbitals with the bond length for the different core-excited states, and we restrict the following discussions only to the states shown in Figure 2a.

In Section 1, we introduced the three groups of energetically well-separated sets of core-excited states denoted as |3d^–1^(5f δ/ϕ)^1^⟩, $|3d^{-1}{\left(5f\pi^{*}\right)}^{1}\rangle$ , and $|3d^{-1}{\left(5f\sigma^{*}\right)}^{1}\rangle$ with either nonbonding 5f ϕ and 5f δ and antibonding 5f π* and 5f σ* valence open-shell orbitals occupied. We start with the discussion of the 5f valence orbital occupation in these six core-excited states and use NSOs for this purpose. The occupation of the 5f valence open-shell orbitals and their variation with the bond length are shown in Figure 5 for *R* = 164–180 pm and in Table 1 for R = 176 pm.

**Figure 5:**
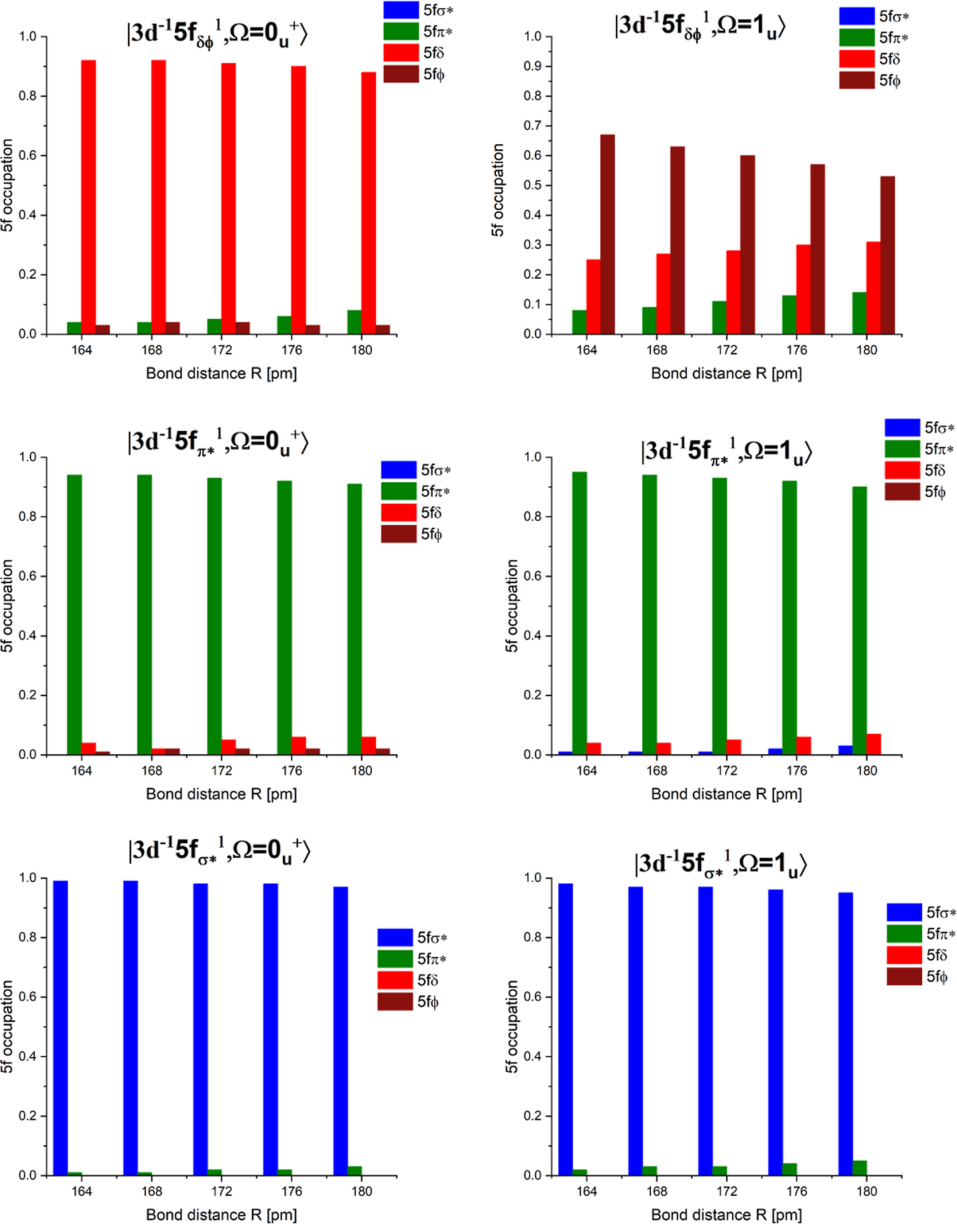
Variation of the occupation of the different 5f valence orbitals in the six core-excited states shown in Figure 2a.

All core-excited states primarily have a 5f occupation, but we provide some additional new information. The core-excited states belonging to the |3d^–1^(5f δ/ϕ)^1^⟩ group can be further subdivided into two subgroups: one group with only 5f δ (>0.88) occupation (see state ${|0_{u}^{+}\rangle }_{III}$ in Table 1) and another group with mixed 5f δ/ϕ occupation (see state ${|1_{u}\rangle }_{V}$ in Table 1). The second subgroup has a pronounced variation of the 5f occupations with varying bond lengths: the 5f ϕ orbitals have the largest occupation, which decreases with longer bond lengths from (0.67 → 0.53). The 5f δ orbitals have substantial occupation as well, but show the opposite trend; they increase with longer bond lengths (0.25 → 0.31), and the same is true for the rather small 5f π* occupation (0.08 → 0.14). All the other groups are dominated by a single 5f π* or 5f σ* occupation (>0.90), respectively, which hardly varies over the considered range of bond lengths. In Table 1, we additionally provide information about the 3d orbital occupation. For the first group of core-excited states, we have the hole located in the 3d δ orbitals, and for the two other groups, the hole is located in the 3d π and 3d σ orbitals.

The valence open-shell orbitals are denoted following the excitation 3d → 5f at uranium as 5f orbitals, which suggests that they are located primarily at uranium. They are formed by antibonding covalent mixing of the U­(5f) and U­(6d) orbitals of the isolated U^6+^ cation and the occupied 2p orbitals of the two O^2–^ anions. In a next step, we study their 5f character, hence the real 5f occupation of these valence orbitals. This information is directly obtained from the projections *N*_p_^val^(5f, |3d^–1^(5fδ/ϕ)^1^⟩,.... (see eq 5). of atomic orbitals of the isolated U­(VI) cation on the 5f valence orbitals of UO_2_^2+^ of the |3d^–1^(5f δ/ϕ)^1^⟩ state and similarly for the other core-excited states. They provide a measure of the extent to which the electron is located either at uranium or at oxygen anions. Along with this, we study their variation with the bond distance, as shown in Figure 6a.

**Figure 6:**
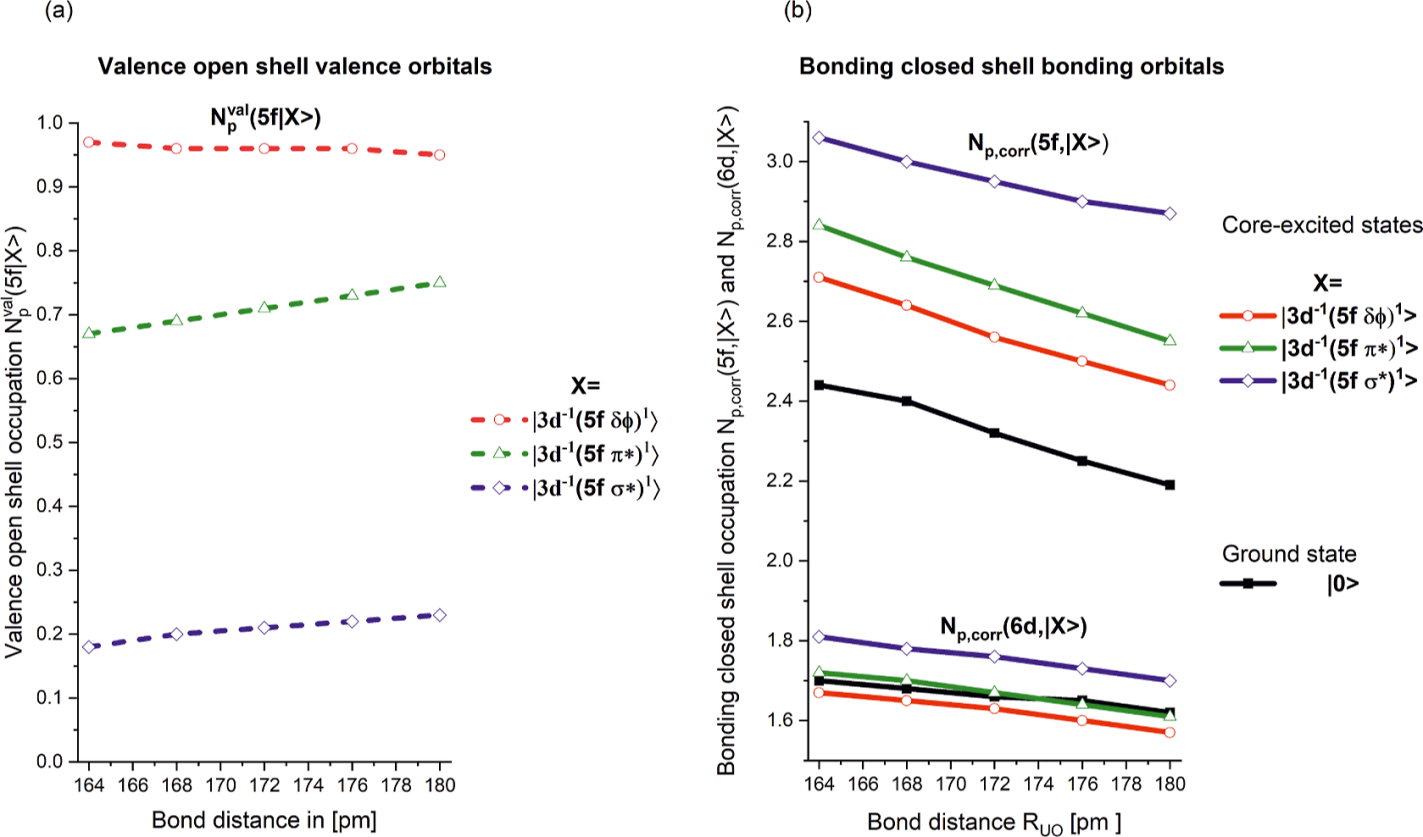
(a) 5f occupation, given by *N*_p_^val^(5f,|3d^–1^(5fδ/ϕ)^1^⟩), $N_{p}^{val}\left(5f,|3d^{-1}{\left(5f\pi^{*}\right)}^{1}\rangle \right)$ and $N_{p}^{val}\left(5f,|3d^{-1}{\left(5f\sigma^{*}\right)}^{1}\rangle \right)$ of the open-shell valence orbitals of the core excited states belonging to the three-branches with 5f δ, ϕ nonbonding or 5f π* and 5f σ* antibonding orbitals occupied. (b) Projection of the 5f and 6d orbitals on the closed-shell orbitals of the ground and core-excited states of uranyl as a function of the bond distance around the equilibrium structure. The corrected occupations *N*_p,corr_(5f, |3d^–1^(5f δ/ϕ)^1^⟩) and *N*_p,corr_(6d, |3d^–1^(5f δ/ϕ)^1^⟩), etc., of uranyl as a function of the bond distance around the equilibrium structure are shown for all states.

For the |3d^–1^(5f δ/ϕ)^1^⟩ states, the 5f valence open-shell occupation is ≈1.0 and hardly changes with the bond length since the occupied nonbonding 5f ϕ and 5f δ orbitals do not covalently mix with oxygen. They are purely atomic uranium orbitals, slightly modified by the presence of the O-anions. This is very different for the two other groups of core excited states, $|3d^{-1}{\left(5f\sigma^{*}\right)}^{1}\rangle$ and $|3d^{-1}{\left(5f\pi^{*}\right)}^{1}\rangle$ , since the occupied antibonding 5f σ* or 5f π* orbitals interact with the 2p orbitals of oxygen. The 5f valence bond open-shell occupations in the $|3d^{-1}{\left(5f\pi^{*}\right)}^{1}\rangle$ and $|3d^{-1}{\left(5f\sigma^{*}\right)}^{1}\rangle$ are ≈0.7 and ≈0.2, respectively. This shows their strong covalent character. Although the notation 5f σ* suggests that this orbital is located at uranium, it has only a 5f occupation of ≈0.2, and therefore, it is primarily of oxygen character. In both cases, the 5f occupation decreases with shorter bond length. This result agrees also very well with the data reported in ref 22.

Now we focus on the bonding closed-shell orbitals and how they differ in two respects: (1) among the core-excited states and (2) from the ground state, as shown in Figure 6b. This is a very important point since it helps to understand how the U­(3d) core-hole is screened by the bonding closed-shell orbitals in the core-excited states. All the corrected values of the closed-shell orbitals for the ground (*N*_p,corr_(5f, |0⟩) and *N*_p,corr_(6d, |0⟩)) and the core-excited states (*N*_p,corr_(5f, |3d^–1^(5f δ/ϕ)^1^⟩), *N*_p,corr_(6d, |3d^–1^(5f δ/ϕ)^1^⟩),···) are shown in Figure 6b. We restrict our discussion to the core-excited states shown with the highest oscillator strengths, as shown in Figure 2a. However, the occupation for the two states for each group, with |0_*u*_^+^⟩ and |1_*u*_⟩, are almost identical in all cases. Therefore, only the results for one of the states are shown for each group.

Overall, the slope of the *N*_p,corr_(5f, |···⟩) and *N*_p,corr_(6d, |···⟩) with the U–O distance is very similar for the ground and the core-excited states. The curves belonging to the 5f and 6d occupations, respectively, are almost parallel to each other (see Figure 6b).

The 6d occupation of the closed-shell orbitals hardly changes between the ground and the three groups of core-excited states. They are virtually identical. This variation is much larger for the 5f occupation. There is a clear order in the 5f occupation of the closed-shell orbitals: $|0\rangle <|3d^{-1}{\left(5f\delta /\phi \right)}^{1}\rangle <|3d^{-1}{\left(5f\pi^{*}\right)}^{1}\rangle <|3d^{-1}{\left(5f\sigma^{*}\right)}^{1}\rangle$ . This indicates that there is more covalent interaction in the $|3d^{-1}{\left(5f\sigma^{*}\right)}^{1}\rangle$ state compared to the other states and a clear ordering for the other states. As for the ground state, the 5f covalent character of the ungerade orbitals is much larger compared to the 6d character in the gerade orbitals, and the increase with decreasing bond length is significantly higher as well for all core-excited states. This is in agreement with our earlier observation^22^ and shows that the screening of the 3d core-hole is by an increase in the 5f occupation.

Complementarily, we investigate the screening of the 3d hole with the 5f valence occupation with the calculation of the $\sum_{i\in g,u}{\left\langle z^{2}\right\rangle }_{i}$ for the orbitals with *g* and *u* symmetry. The spatial extent of the sums over the g and u orbitals provides information about covalent mixing of the oxygen anions with U­(6d) and U­(5f), respectively. Figure 4a,b shows that all the different $\sum_{i\in g,u}{\left\langle z^{2}\right\rangle }_{i}$ , for the ground and all the core-excited states, increase significantly as the U–O distance increases.

The changes between the sizes of the charge distribution of the closed-shell orbitals for the ground state and the various core-excited states provide direct information about the contraction of the orbitals due to the 3d core-hole. This was extensively discussed in ref 22 here we focus on the differences between the ground and the core-excited states. The sizes of the charge distributions of the gerade states are almost on top of each other and change (see Figure 4a) much less compared to the ungerade states (see Figure 4b) when comparing the ground state with the core-excited states. The absolute values of the $\sum_{i\in g}{\left\langle z^{2}\right\rangle }_{i}$ for the g symmetry hardly vary for the ground and all the core-excited states. In contrast to that the $\sum_{i\in u}{\left\langle z^{2}\right\rangle }_{i}$ for the u symmetry are reduced considerably compared to the results for the ground state. This shows that the bonding closed-shell orbitals with ungerade symmetry contract significantly more and therefore contribute more to the screening of the 3d hole compared to the orbitals with gerade symmetry.

The slopes of the sum over gerade orbitals hardly differ between the ground and all three groups of core-excited states. Hence there are only minor changes in the electronic structure of the gerade orbitals between the ground- and core-excited states. In contrast to that the slopes of the sum over ungerade orbitals change significantly between the ground and the core excited states. The slopes for all core-excited states are much smaller compared to the ground state and decrease in the order $|0\rangle >|3d^{-1}{\left(5f\delta /\phi \right)}^{1}\rangle \approx |3d^{-1}{\left(5f\pi^{*}\right)}^{1}\rangle >|3d^{-1}{\left(5f\sigma^{*}\right)}^{1}\rangle$ . This indicates that there is more covalent interaction in the ungerade orbitals and the $|3d^{-1}{\left(5f\sigma^{*}\right)}^{1}\rangle$ core-excited state has the most covalent character.

This result is in total agreement with the previous result for the projections (see Figure 6b) where we found that there is a considerable increase in the 5f occupation in the $|3d^{-1}{\left(5f\sigma^{*}\right)}^{1}\rangle$ state. Both measures also give the same order for the covalent interaction. This shows that both measures provide consistent information about the changes in the electronic structure required to screen the 3d core-hole.

### 3.3 Simulation of the UO_2_^2+^ U M_5_ Absorption Edge XANES for Varying Bond Lengths

A first application of the potential energy curves to spectroscopy are the study of the changes of the UO_2_^2+^ U M_5_ absorption edge XANES with the bond length. For the simulation of the UO_2_^2+^ U M_5_ absorption edge XANES, we broaden the calculated dipole intensities with a Voigt convolution^53^ of a Gaussian with Full Width at Half Maximum, FWHM, of 1.5 eV and a Lorentzian with FWHM of 3.5 eV for the M_5_ lifetime. The Gaussian FWHM was chosen as a very rough measure of the experimental resolution for the specific geometry of the measurements. In particular, it also provided a rather good fit, as we show below, between our theory and the experimentally derived curves. The Lorentzian FWHM for lifetime of an M_5_ hole in U was taken from the compendium of Campbell and Papp.^54^

In Figure 7, we show the simulated UO_2_^2+^ U M_5_ absorption edge XANES at the reported experimental assumed equilibrium structure of 176 pm.^30,31^ Along with the total XANES spectrum, we give the contributions from the three different groups of core-excited states and compare with the experimentally available HR-XANES data,^16,22^ which is shown in Figure 7 as a gray dotted line. For more details about the HR-XANES data, see ref 16. The total simulated XANES spectrum, which should be compared with the experiment, is shown in black. The contributions from the excitations into the |3d^–1^(5f δ/ϕ)^1^⟩ and |3d^–1^(5f δ)^1^⟩ subgroups are shown in red and orange, respectively. The other two groups, $|3d^{-1}{\left(5f\pi^{*}\right)}^{1}\rangle$ ) and $|3d^{-1}{\left(5f\sigma^{*}\right)}^{1}\rangle$ , are shown in green and blue, respectively. Along with this, we give the energies and oscillator strength of the most intense excitations for each group (see Table 1) as vertical bars in Figure 7.

**Figure 7:**
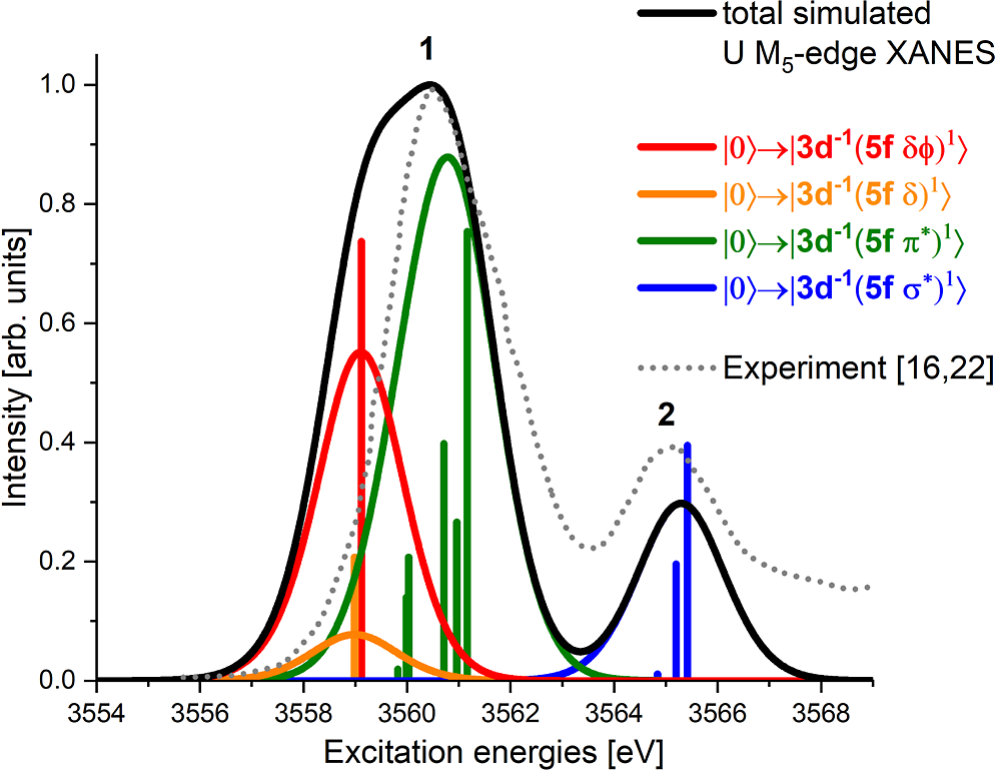
Simulated UO_2_^2+^ U M_5_ absorption edge XANES for *R* = 176 pm shifted by Δ*E* = 16.00 eV. The experimental HR-XANES data are reported in ref 16,22 . The experimental spectrum was from Figure 2 in ref 22.

The total simulated XANES spectrum agrees very well with the experimental result^16,22^ apart from a constant small shift of 16.00 eV. The overall shape with only two pronounced peaks, labeled as peak 1 and **2** in Figures 7 and 8a, is reproduced with our simulations. Peak 1 is formed by excitations into the |3d^–1^(5f δ/ϕ)^1^⟩, |3d^–1^(5f δ)^1^⟩ and $|3d^{-1}{\left(5f\pi^{*}\right)}^{1}\rangle$ ) groups of core-excited states, whereas peak **2** can be assigned to excitations into the $|3d^{-1}{\left(5f\sigma^{*}\right)}^{1}$ group. The experimentally reported peak splitting of 4.81 eV is very close to our theoretical result of 4.75 eV, which emphasizes the high quality of the calculations. The first peak in the experimental data is clearly narrower compared to the simulation because (1) we compare a conventional XANES simulation with a cut through the RIXS map, which displays narrower line broadening, and (2) the shape of the simulation critically depends on the calculated excitation energies and the oscillator strengths. Even small errors in the calculated data can cause large changes in the simulation. In our cases these are the relative excitation energies into core-excited states of the $|3d^{-1}{\left(5f\delta /\phi \right)}^{1}\rangle$ and $|3d^{-1}{\left(5f\pi^{*}\right)}^{1}\rangle$ groups and the corresponding oscillator strengths.

**Figure 8:**
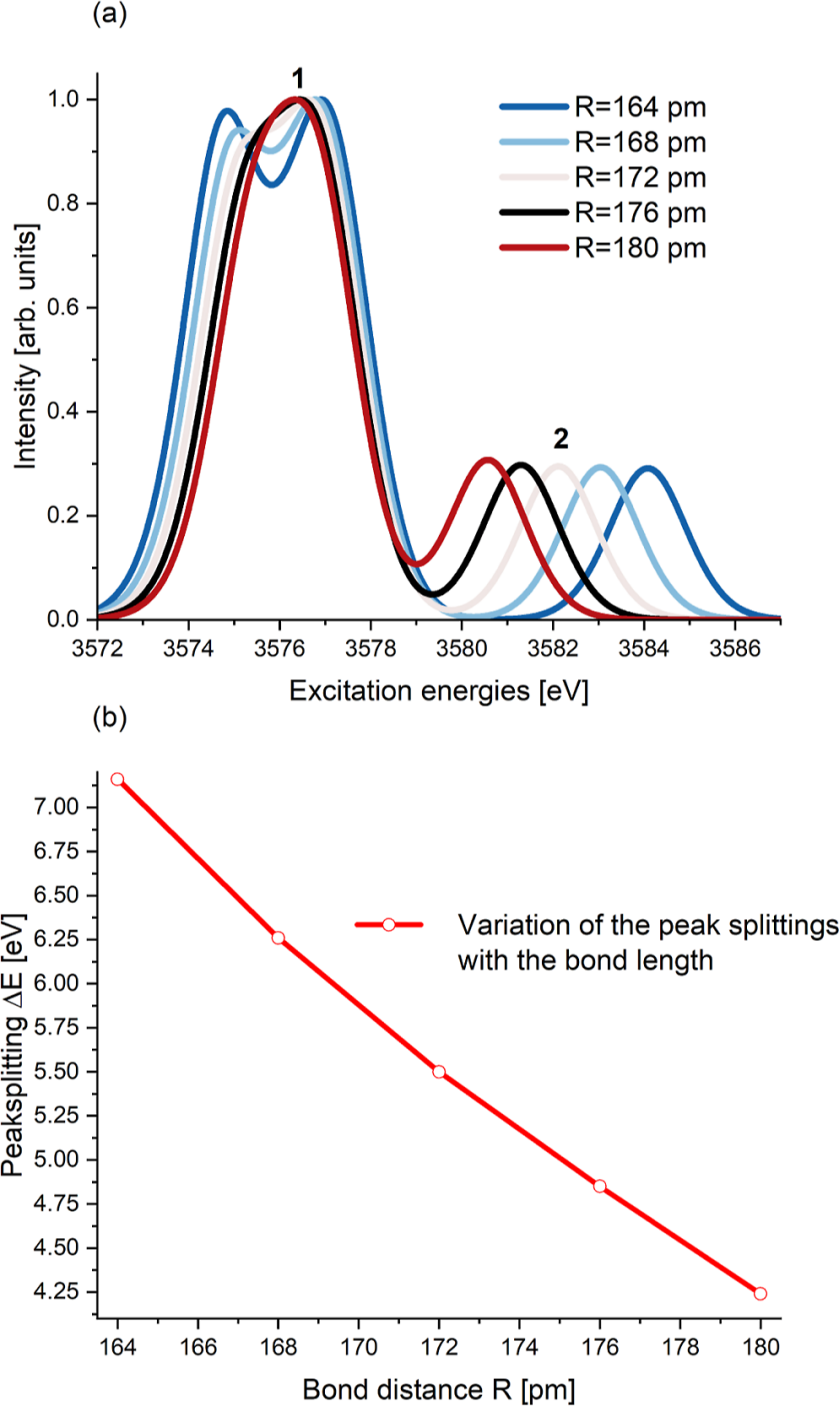
(a) Simulated UO_2_^2+^ U M_5_ absorption edge XANES for *R* = 164–180 pm. (b) Variation of the peak splittings of the simulated spectra shown in (a) between the two main peaks.

Additionally, the shape of the U M_5_ absorption edge of XANES can be nicely explained by the various contributions. Experimentally^16,22^ there are only two peaks, which is different from the U M_4_ absorption edge XANES, which shows three distinct peaks. The reason for this is that in the U M_5_ edge XANES, the excitations of the first two groups (|3d^–1^(5f δ)^1^⟩/|3d^–1^(5f δ/ϕ)^1^⟩ (shown in orange and red) and $|3d^{-1}{\left(5f\pi^{*}\right)}^{1}\rangle$ (shown in green) are energetically closer to each other and that there are many more intense contributions from the excitations into the $|3d^{-1}{\left(5f\pi^{*}\right)}^{1}\rangle$ group, as can be seen from Figure 7. Additionally, the excitations into the first group of core-excited states have less intensity relative to the second group.

The assignment given here is very similar to the assignment in ref 22. The excitation scheme used in this work corresponds to the Open-Shell Active (OSA) scheme; excitations as in the Open Closed-Shell Active (OCSA) are not included. The simulated OSA and OCSA U M_5_ absorption edge XANES show all three features in contrast to the two we find in this work. In this work, we used a slightly larger Gaussian broadening, primarily because the experimental measurement could be better reproduced. As mentioned above, the intensities of the two maxima of the first feature are much closer to each other in this work compared to the OSA and OCSA results presented in ref 22 and therefore form only a single broad peak. The simulated XANES spectra are all shifted by 16.00 eV compared to the spectra presented in Figure 1 (OSA) and 2 (OCSA) in ref 22. The reason for this is the difference in the theoretical approaches, as explained in Section 2.

In Figure 8a we show the simulated UO_2_^2+^ U M_5_ absorption edge XANES for different bond distances *R* = 164–180 pm. There is significant variation of the simulated U M_5_ edge XANES with increasing bond length. The position of the first broad peak hardly changes, but the position of the second peak moves very systematically with the bond length. Additionally, for *R* ≤ 172 pm, peak 1 splits up into two sub peaks. We use the position of the slightly higher intensity sub peak, at the excitation energy slightly above 3576 eV, as the position of peak 1. The maxima move energetically closer to each other with an increasing bond length. This can be explained by the shapes of the potential energy curves. Therefore, a very systematic variation is seen for the peak splittings between the first and second peak in the UO_2_^2+^ U M_5_ absorption edge XANES.

In Figure 8b, we show the calculated peak splittings of the XANES spectra shown in Figure 8a. The peak splitting varies almost linearly with the bond distance, and the reason for this is the shape of the potential energy curves of the core-excited states. This agrees with experimental findings for the U M_4_ absorption edge XANES reported by Amidani et al.^4^ that for a large class of uranyl systems the peak splittings correlate linearly with the bond distances.

The main conclusion here is that the choice of the structure for calculation of an XANES spectrum can lead to significant differences in the predicted spectra. In addition, we caution that the requirements for a correct theoretical description of the satellite XANES features are considerably greater than for the most intense, main XANES features.

## 4 Establishing a Link between X-ray Spectroscopy and the Chemical Bonding

In Section 3.1.1, we quantified the covalency in the ground state due to the interaction of the 5f orbitals of uranium with the 2p orbitals of oxygen. This is summarized in Figure 3, where we give the variation of the 5f character with the bond length for the ground state and found a monotonic, almost linear, decrease of the 5f occupation with increasing bond length. In Section 3.3, we simulated U M_5_ edge XANES for different U–O distances, which gives us access to the peak splittings with varying bond lengths (see Figure 8b). In this case, we found an almost linear decrease of the peak splitting with the bond length as well. In Figure 9, we combine these data sets and get direct information on how the peak splitting in the UO_2_^2+^ U M_5_ absorption edge XANES and the covalent character are correlated. It is obvious that there is only a very small variation of the 5f occupation in the bonding closed-shell orbitals of the ground state given by *N*_p,corr_(5f, |0⟩) with peak splitting. The peak splitting varies by a factor of 1.68, compared to a rather small variation of 1.11 of the 5f occupation.

**Figure 9:**
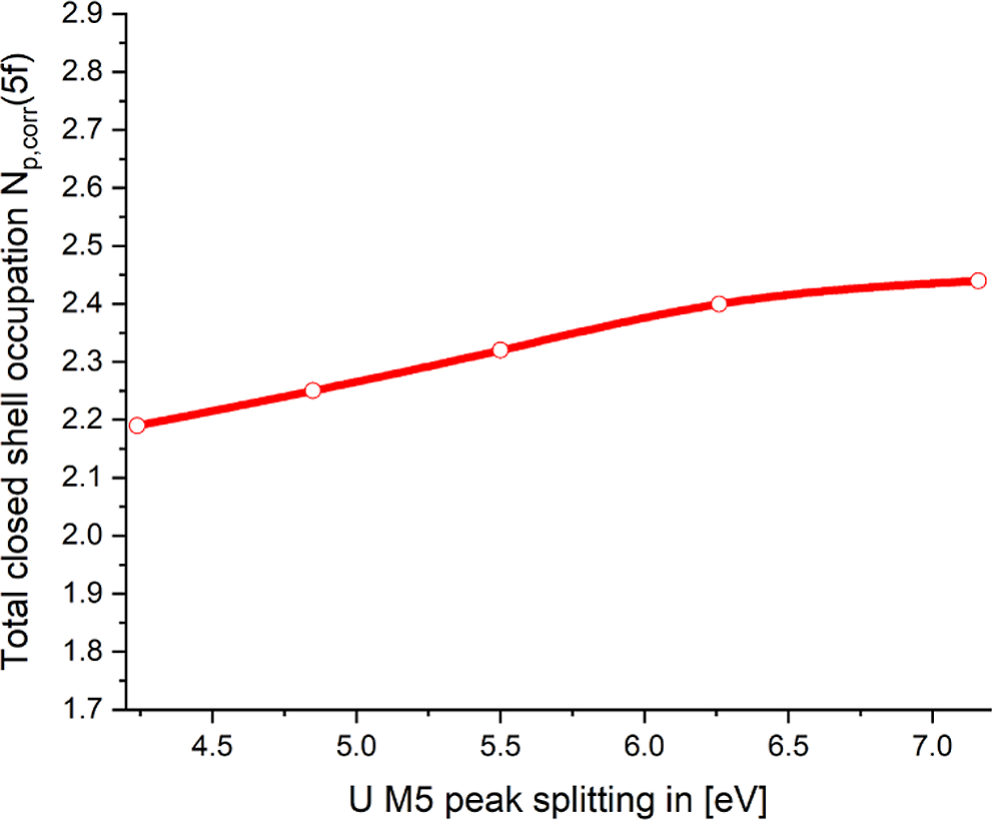
Dependence of the projections *N*_p,corr_(5f, |0⟩) of the ground state on the peak splittings of the UO_2_^2+^ U M_5_ absorption edge XANES.

For a large class of uranyl compounds, the U–O bond distances range from 176 to 181 pm.^4^ This restricts the range of peak splittings which are of interest to ≈4.2–4.9 eV. Hence, in the range which is of chemical interest, we can expect a variation of the peak splittings by a factor of 1.167, which goes along with a modest increase of the 5f occupation by only ≈1.026.

With this we can clearly establish a link of the results for the UO_2_^2+^ U M_5_ absorption edge XANES with the chemistry of uranyl and quantify how covalency in the uranyl bond is varying with the measured peak splitting in the XANES spectra. Additionally, we confirm the correlation of the peak splittings with the covalency postulated by Vitova et al.^21^ and extend their work by considering the covalent character as a function of the bond distance.

## 5 Conclusion

We studied the ground- and core-excited states of the U M_5_ absorption edge manifold of uranyl and determined their potential energy curves. We restricted this discussion to the core-excited states with dipole-allowed transitions (|0_*g*_^+^⟩ → |0_*u*_^+^⟩ and |0_*g*_^+^⟩ → |1_*u*_⟩) from the ground state with the highest oscillator strengths between the ground and the core-excited states and provided the spectroscopic parameters of these states. The differences in the shapes of these curves are discussed, and the variation of the covalent character of the ground and the core-excited states is studied with two independent measures.

In the ground state, the covalent mixing of the oxygen orbitals with the 5f orbitals of uranium is much more pronounced compared to the mixing with the 6d orbitals. With the determination of the projections, we can quantify the change in the covalency in the ground state with bond length. The calculations of *∑*_*i*∈*g*,*u*_⟨*z* ^2^⟩ of the closed-shell orbitals in the ground state provide additional consistent information. The slope of the sum over ungerade orbitals is smaller compared to the slope of the sum over gerade orbitals, indicating that there is more covalent interaction of the U­(5f) orbitals with the oxygen anions compared to the covalent interaction of the U­(6d) orbitals.

For the core-excited states, we report spectroscopic constants of the states as well and observe considerable changes for the equilibrium distance and the frequencies compared to the ground state. The occupied 5f open-shell valence orbitals are characterized using NSOs and their 5f character is determined. The determination of the projections of the U­(5f) and U­(6d) orbitals of the isolated U^6+^ cation on the closed-shell bonding orbitals of the uranyl molecule, UO_2_^2+^ allow to determine the extent of the covalent 5f and 6d character in the bonding closed-shell orbitals of all these states. For all core-excited states, the 5f character is substantially increased whereas the changes in 6d character compared to the ground state are rather small. The reason for this is the screening of the 3d hole by the increase of the 5f occupations in the bonding orbitals. This is confirmed considering the spatial extent of the gerade and ungerade orbitals using the sums *∑*_*i*∈*g*,*u*_⟨*z* ^2^⟩. They indicate increasing 5f covalency in the core-excited states compared to the ground state in the same order as determined by the projections. Moreover, looking at the slopes of the sums, this measure shows that there is more covalent interaction in the ungerade orbitals due to the interaction of the U­(5f) orbitals with the oxygen anions compared to the covalency in the gerade states due to the covalent mixing with the U­(6d) orbitals.

In our recent work,^22^ and here, we studied measures which are indicative of the covalency of the uranyl bond and simulated the U M_5_ edge XANES spectra. The 4-component fully relativistic approach and the approach in this work are based on the second-order Douglas–Kroll–Hess (DKH) Hamiltonian^27,28^ with spin–orbit effects included by perturbation theory^29^ provide a consistent picture. The simulated XANES spectra differ slightly, but overall, the different features and characteristics are described in a very similar way. The same is true for the two measures of covalency.

The theoretical model employed in this work and our recent work^22^ are essential for explaining all the features in the experimental spectrum.^16,22^ The uranyl UO_2_^2+^ U M_5_ absorption edge, 3d_5/2_ → 5f_5/2_ and 3d_5/2_ → 5f_7/2_, XANES spectra are formed by many unresolved excitations to core-excited states that have nearly the same energy.^15,16^ But due to the lifetime broadening, they cannot be resolved individually. Only the calculated spectra allow a detailed understanding and interpretation of the experimental spectra^16,22^ and how they are formed by the individual transitions.

Combining the spectroscopic results from our calculation with the information about the electronic structure and the covalency, we can clearly establish a link between results from the UO_2_^2+^ U M_5_ absorption edge XANES with the chemical bonding of uranyl and quantify how covalency in the uranyl bond is varying with the measured peak splitting in the XANES spectra. Hence, the correlation of the peak splittings with the covalency postulated by Vitova et al.^21^ is confirmed and extended by considering the variation of the covalent character with the bond distance and thereby quantifying the correlation of the peak splittings with the covalent character.

## Supplementary Material



## Notes

Schematic of the ideal 5f orbitals of an isolated actinide cation and additional information about the applied theoretical methods (PDF)
