# Supplementary material for: Potential Energy Curves of Core-Excited States of the U M5 Absorption Edge Manifold of UO2 2+
Source: Inorg Chem. 2026 Feb 19;65(9):4925–38. doi: 10.1021/acs.inorgchem.5c04776 (PMC12977062; doi:10.1021/acs.inorgchem.5c04776)
Supplement: Supplementary file 1 [file ic5c04776_si_001.pdf]

Potential Energy Curves of Core-Excited States of the U M_5 absorption edge manifold of UO_2^{2+} - Supporting Information

Robert Polly*,† and Paul Bagus*,‡

†*Karlsruher Institut für Technologie (KIT), Campus Nord, Institut für Nukleare Entsorgung (INE), Hermann von Helmholtzplatz 1, 76344 Eggenstein-Leopoldshafen, Germany*

‡*Department of Chemistry, University of North Texas, Denton, Texas 76203-5017, United States*

E-mail: polly@kit.edu; Paul.Bagus@unt.edu

January 7, 2026

S1 Supporting information

S1.1 Schematic of the ideal 5f orbitals of an isolated actinide cation

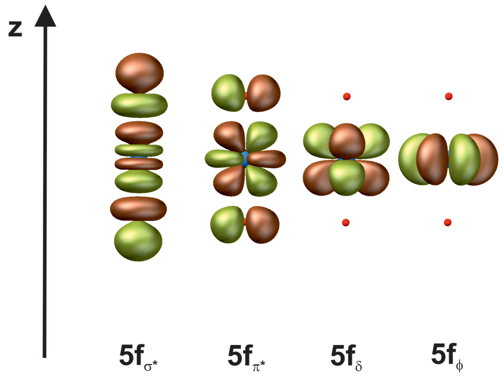

Figure S1: Schematic of the ideal 5f orbitals of an isolated actinide cation. In an actinyl, the z axis is the the position of the O anions, above and below, the cation.

S1.2 Methods

The choice of the active spaces in the HR-XANES is the same as in[1] The five *3d* core orbitals form the RAS1 active space and the seven *5f* orbitals forming either non-bonding or anti-bonding orbitals with the oxygen *2p* are placed in the RAS3 active space. The RAS2 active space is kept empty. We allow two holes in RAS1 and two electrons in RAS3. This active space is designated $3d/5f$ and adequately describes the electronic structure of the U M_5 absorption edge manifold of uranyl with one hole in the *3d* shell and one *5f* valence orbital occupied. The difference to other recent studies[2–5] is that additional, many electron, excitations from the bonding orbitals to the valence orbitals of uranyl are not considered in this work. our previous calculations where the state averaging in the RASSCF/RASPT2 calculations was performed over all the core-excited states $|3d^{-1}5f^1\rangle$, with singlet (35 states) and triplet (35 states) spin, together, in one RASSCF/RASPT2 calculation.

Therefore we did three separate calculations of the three different sets of core-excited states by restricting the state-averaging to only one set of core-excited states, respectively. With this approach the orbitals are optimized specifically for the three different sets. In the first set $|3d^{-1}(5f\,\delta/\phi)^1\rangle$, there are four *5f* valence orbitals occupied with one hole in the $3d$ core electron shell. Therefore we have 20 core-excited states of the first set. Similarly 10 of the second and 5 of the third set for $S = 0$ and $S = 1$, each (summing up to 35 in both cases). This allows also to investigate and characterize the closed shell $\sigma_{u,g}$ and $\pi_{u,g}$ orbitals of the three sets of core-excited states separately.

S2 References

References

(1) Polly, R.; ; Schacherl, B.; Rothe, J.; Vitova, T. Relativistic Multiconfigurational Ab Initio Calculation of Uranyl 3d4f Resonant Inelastic X-ray Scattering. *Inorganic Chemistry* **2021**, *60 (24)*, 18764.

(2) Stanisstreet-Welsh, K.; Kerridge, A. Bounding $[AnO_2]^{2+}$ (An = U, Np) covalency by simulated O K-edge and An M-edge X-ray absorption near-edge spectroscopy. *Physical Chemistry Chemical Physics* **2023**, *25*, 23753.

(3) Bagus, P. S.; Nelin, C. J.; Rosso, K. M.; Schacherl, B.; Vitova, T. Electronic Structure of Actinyls: Orbital Properties. *INORGANIC CHEMISTRY* **2024**, *63*, 1793–1802.

(4) Ehrman, J. N.; Shumilov, K.; Jenkins, A. J.; Kasper, J. M.; Vitova, T.; Batista, E. R.; Yang, P.; Li, X. Unveiling Hidden Shake-Up Features in the Uranyl M4-Edge Spectrum. *JACS* **2024**,

(5) Bagus, P. S.; Nelin, C. J.; Schacherl, B.; Vitova, T.; Polly, R. Bonding and Interactions in UO_2^{2+} for Ground and Core Excited States: Extracting Chemistry from Molecular Orbital Calculations. *JOURNAL OF PHYSICAL CHEMISTRY A* **2024**, *128*, 8024–8034.
